# Effect of different positive end expiratory pressure levels on optic nerve sheath diameter in patients with or without midline shift who are undergoing supratentorial craniotomy

**DOI:** 10.1007/s00701-024-06067-1

**Published:** 2024-04-15

**Authors:** Adem Fenerci, Eren Fatma Akcil, Yusuf Tunali, Ozlem Korkmaz Dilmen

**Affiliations:** Department of Anaesthesia & Intensive Care, Cerrahpasa Medical Faculty, Istanbul University-Cerrahpasa, University of Istanbul, 34098 Cerrahpasa Istanbul, Turkey

**Keywords:** Craniotomy, Intracranial pressure, Elevated, Positive end expiratory pressure, Optic nerve sheath

## Abstract

**Purpose:**

In general, high levels of PEEP application is avoided in patients undergoing craniotomy to prevent a rise in ICP. But that approach would increase the risk of secondary brain injury especially in hypoxemic patients. Because the optic nerve sheath is distensible, a rise in ICP is associated with an increase in the optic nerve sheath diameter (ONSD). The cutoff value for elevated ICP assessed by ONSD is between 5.6 and 6.3 mm. We aimed to evaluate the effect of different PEEP levels on ONSD and compare the effect of different PEEP levels in patients with and without intracranial midline shift.

**Methods:**

This prospective observational study was performed in aged 18–70 years, ASA I–III, 80 patients who were undergoing supratentorial craniotomy. After the induction of general anesthesia, the ONSD’s were measured by the linear transducer from 3 mm below the globe at PEEP values of 0–5–10 cmH_2_O. The ONSD were compered between patients with (*n* = 7) and without midline shift (*n* = 73) at different PEEP values.

**Results:**

The increases in ONSD due to increase in PEEP level were determined (*p* < 0.001). No difference was found in the comparison of ONSD between patients with and without midline shift in different PEEP values (*p* = 0.329, 0.535, 0.410 respectively). But application of 10 cmH_2_O PEEP in patients with a midline shift increased the mean ONSD value to 5.73 mm. This value is roughly 0.1 mm higher than the lower limit of the ONSD cutoff value.

**Conclusions:**

The ONSD in adults undergoing supratentorial tumor craniotomy, PEEP values up to 5 cmH_2_O, appears not to be associated with an ICP increase; however, the ONSD exceeded the cutoff for increased ICP when a PEEP of 10 cmH_2_O was applied in patients with midline shift.

## Introduction   

Dealing with hypoxemia in patients with intracranial pathology and elevated intracranial pressure would be challenging in clinical practice. Application of positive end expiratory pressure (PEEP) increases mean airway pressure, prevents alveolar collapse, and improves lung compliance and oxygenation [15]. In general, high levels of PEEP application is avoided in patients undergoing craniotomy to prevent a rise in intracranial pressure (ICP). But that approach would increase the risk of secondary brain injury especially in patients with low lung compliance and hypoxemia. Recent studies showed that if the patients are euvolemic and normotensive, PEEP application may not cause a rise in ICP [14]. The basic indications of PEEP application are reduction in functional residual capacity and lung compliance as well as hypoxemia [7].

The optic nerve sheath is in direct continuity with the dura mater and cerebrospinal fluid (CSF). Because the optic nerve sheath is distensible, a rise in ICP is associated with an increase in the optic nerve sheath diameter (ONSD) [12, 13]. The reported optimal ONSD cutoff values in the literature are heterogeneous, and no consensus exists about the definitive ONSD to diagnose elevated ICP. However, Berhanu et al. [2] comprised a total of 2824 patients in their meta-analysis and showed that ONSD cutoff values of 5.6 to 6.3 mm were associated with higher pooled specificity and no loss of sensitivity compared to cutoff values of 4.9 to 5.5 mm.

Therefore, we aimed to evaluate the effect of different PEEP levels on ONSD in patients undergoing supratentorial craniotomy. As one of the important indicators of elevated intracranial pressure is presence of intracranial midline shift on neuro images, we also compared the effect of different PEEP levels on ONSD in patients with and without intracranial midline shift.

## Methods

The Ethics Committee of Istanbul University-Cerrahpasa, Cerrahpasa School of Medicine, Istanbul, Turkey (Chairperson Prof Ozgur Kasapcopur), provided ethical approval for this study on 24 February 2021 (Ethical Committee No: 37288).This prospective and observational study was performed during 1 year period after the ethics committee approval in Istanbul University-Cerrahpasa, Cerrahpasa School of Medicine, Neurosurgical Operation Rooms. After written informed consent, 82 ASA I–III patients aged between 18 and 70 years scheduled for elective supratentorial craniotomy were included in the study.

Patients presenting with intracranial herniation, hemodynamic instability, and hypotension (mean arterial pressure < 55 mmHg) during PEEP application were excluded from the study.

In the operating room, with routine ASA monitoring, bispecteral index (BIS) and proceeded electroencephalography monitoring were performed. Anesthesia was induced with propofol (the dose was titrated until delta waves were observed on EEG), rocuronium (0.5 mg kg^−1^), and remifentanil (0.1 µg kg^−1^ min^−1^). After 2 min of manual ventilation with oxygen/air (FiO_2_ = 0.8), patients were intubated and ventilated with volume-controlled mode, tidal volume 8 mL kg^−1^ (ideal body weight), FiO_2_ = 0.4, inspiration:expiration ratio of 1:2, and PEEP 5 cmH_2_O, and the respiratory rate (9–12 per minute) was adjusted to maintain PaCO_2_ in the range of 36 to 38 mmHg. Anesthesia was maintained with remifentanil (0.05–0.15 µg kg^−1^ min^−1^) and sevoflurane (0.8–1 MAC in oxygen/air).

**Fig. 1:**
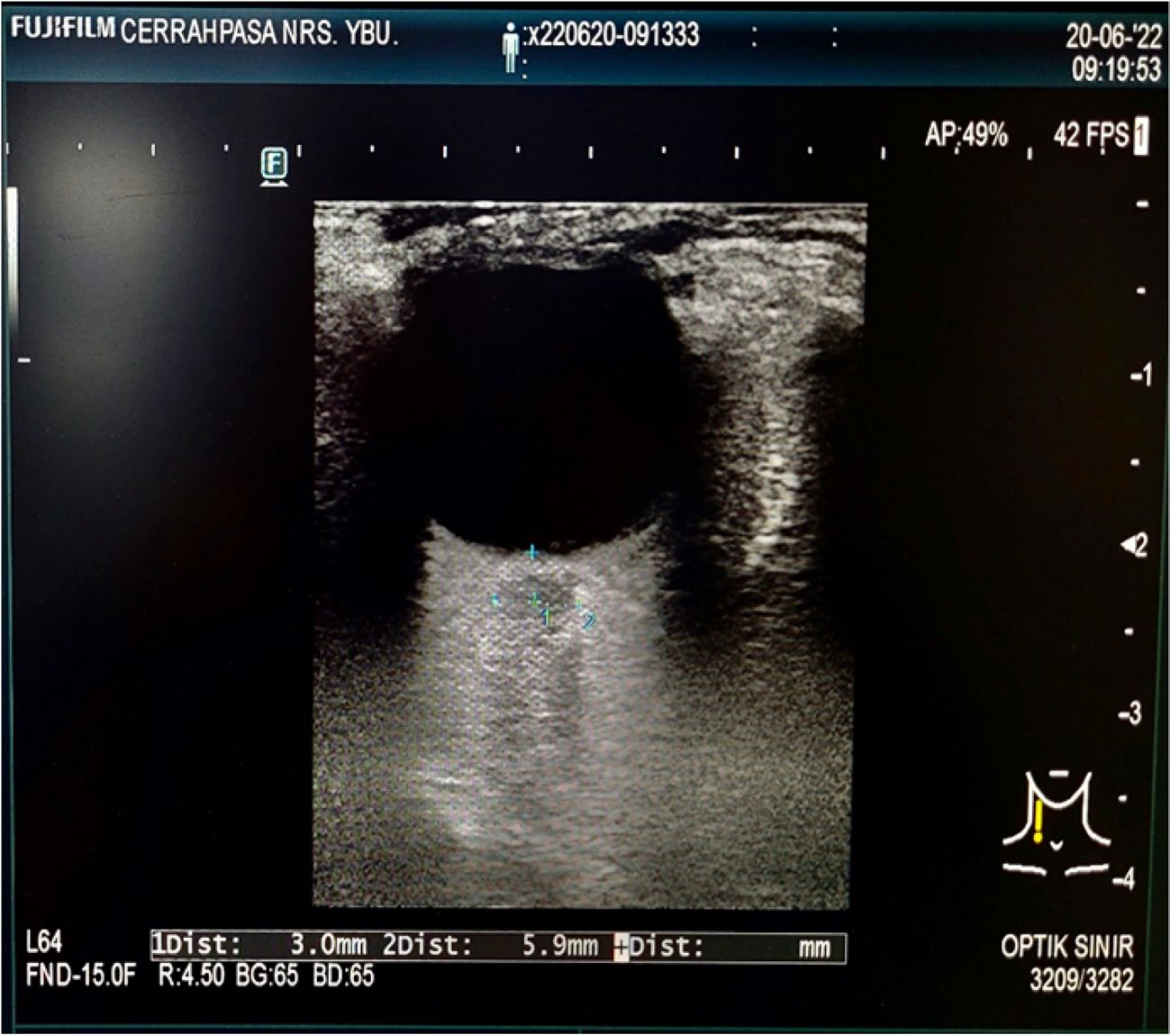
Ultrasonography guided OSND measurement

After intra-arterial blood pressure monitoring, the ONSD was measured by the linear transducer from 3 mm below the globe at PEEP values of 0, 5, and 10 cmH_2_O respectively (ARIETTA 50 USG device). The ONSD measurement was performed on the eye with the best image quality. The linear transducer was applied with coupling gel to the closed eyelid (Fig. 1). The PEEP value was adjusted at the level of 0 cmH_2_O and the ONSD was measured from 3 mm below the globe at expiration in maximum 30 s. After that, the PEEP value was increased up to 5 cmH_2_O and following 5-min ventilation with that PEEP value the ONSD was measured again. Then the PEEP value increased to 10 cmH_2_O and the ONSD measurement was repeated after 5-min ventilation. The PEEP application was stopped if mean arterial pressure reduced below 55 mmHg. Besides this, the peak airway pressures were measured in 5-min intervals.

We defined a cutoff for the ONSD of 5.6 mm to indicate the transition between “normal” and “increased” ICP.

The presence of midline shift was determined by checking of the cranial MRI or CT scans before surgery.

### Statistical analysis and sample size

The power analysis was performed with the G*Power statistical program (ver.3.1.9.7). A total of 54 patients were calculated for a Cohen’s *d* effect size of 0.5 (which translates to 0.25-mm minimum difference when a mean 4.75 mm and a standard deviation of 0.5 mm is accepted for baseline OND) with a probability error type I of 0.05 and power of 0.95. We enrolled 80 patients according to this: “in one sample *t*-test experimental design”; when *n* is 80, effect size 0.5 (*t*-test effect size average value) and type-1 error (α) 0.05; “Power was found to be 99% [6]” (Table 1).

**Table 1:** Power analysis

*t* tests—Means: Difference from constant (one sample case)	
Analysis:	Post hoc: Compute achieved power
Input:	Tail(s) = Two
	**Effect size *d* = 0.5**
	**α err prob = 0.05**
	**Total sample size = 80**
Output:	Noncentrality parameter δ = 4.4721
	Critical *t* = 1.9904
	Df = 79
	**Power (1-β err prob) = 0.9930**

All data were expressed as a number or mean (SD). The SPSS for Windows, ver.26, was used for statistical analysis. The Kolmogorov–Smirnov test was used to evaluate the distribution of data. The variables were normally distributed among the groups; therefore, repeated measures of ANOVA by Bonferroni correction were used within group comparisons and independent *t* test was used among the group comparisons. *p* < 0.05 was considered to be statistically significant.

## Results

**Fig. 2:**
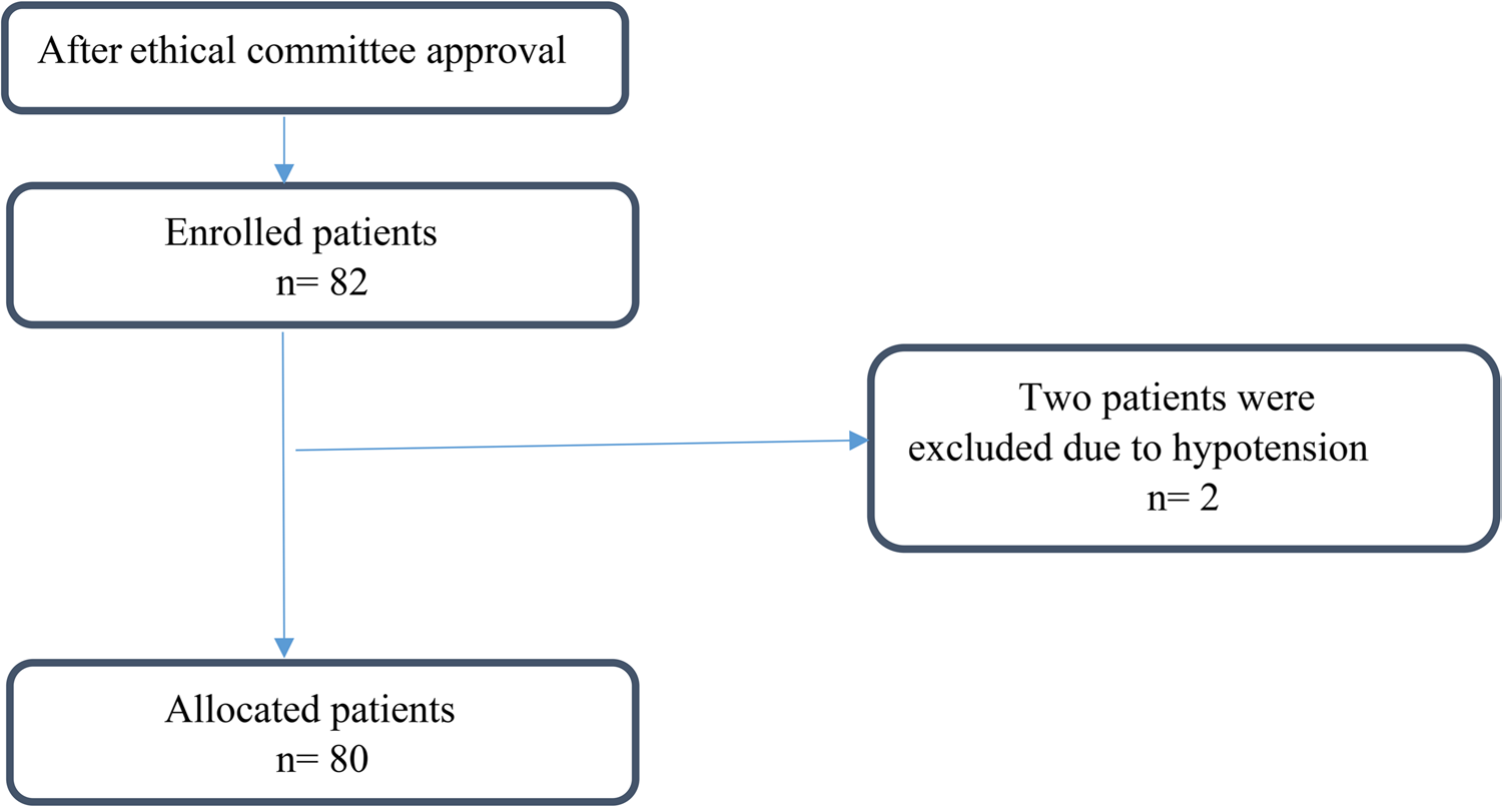
Flow chart

Eighty-two patients were enrolled in this study. Two patients were excluded from the study due to presenting hypotension during 10 cmH_2_O PEEP application. Total 80 patients allocated to the study (Fig. 2).

Patients’ demographic values and diagnoses are shown in Table 2.

**Table 2:** Patients’ demographic values and diagnoses

	*n* = 80
ASA I/II/III (n)	32/42/6
Gender, M/F (n)	28/52
Age, year (mean ± SD)	45.96 ± 16.33
Body weight, kg (mean ± SD)	77.21 ± 16.28
Height, cm (mean ± SD)	166.75 ± 8.08
Body mass index (mean ± SD)	27.67 ± 5.64
Pathology (*n*)	
Supratentorial tumor	62
Aneurysm	8
Arteriovenous malformation	6
Epilepsy	4

*ASA* American Society of Anaesthesiologists, *BMI* body mass index, *n* number

Statistical significant increase was observed in the ONSD due to increase in PEEP levels in the study population (*p* < 0.001, Table 3).

**Table 3:** Effect of different positive end expiratory pressure levels on optic nerve sheath diameter in all patients

	*Mean* ± *SD*	*Minimum*	*Maximum*	*p
*ONSD when PEEP 0 cmH*_*2*_*O (mm)*	*4.77* ± *0.49*	*3.70*	*6.10*	
*ONSD when PEEP 5 cmH*_*2*_*O (mm)*	*5.10* ± *0.58*	*3.90*	*7.00*	*0.001*
*ONSD when PEEP 10 cmH*_*2*_*O (mm)*	*5.44* ± *0.71*	*4.00*	*7.70*	

^*^Repeated measures of ANOVA with the post hoc Bonferroni correction test

No statistically significant difference was found in the comparison of ONSD between patients with and without midline shift in different PEEP values (*p* = 0.329, 0.535, 0.410 respectively, Table 4). Application of 10 cmH_2_O PEEP in patients with a midline shift increased the mean ONSD value to 5.73 mm (Table 4).

**Table 4:** Comparison effect of different positive end expiratory pressure levels on optic nerve sheath diameter in patients with or without midline shift

	*With midline shift (n* = *7)* *Mean* ± *SD*	*Without midline shift (n* = *73)* *Mean* ± *SD*	*p
*ONSD when PEEP 0 cmH*_*2*_*O (mm)*	5.00 ± 0.14	4.76 ± 0.49	0.329
*ONSD when PEEP 5 cmH*_*2*_*O (mm)*	5.28 ± 0.15	5.09 ± 0.60	0.535
*ONSD when PEEP 10 cmH*_*2*_*O (mm)*	5.73 ± 0.25	5.42 ± 0.72	0.410

^*^Independent *t*-test

**Fig. 3:**
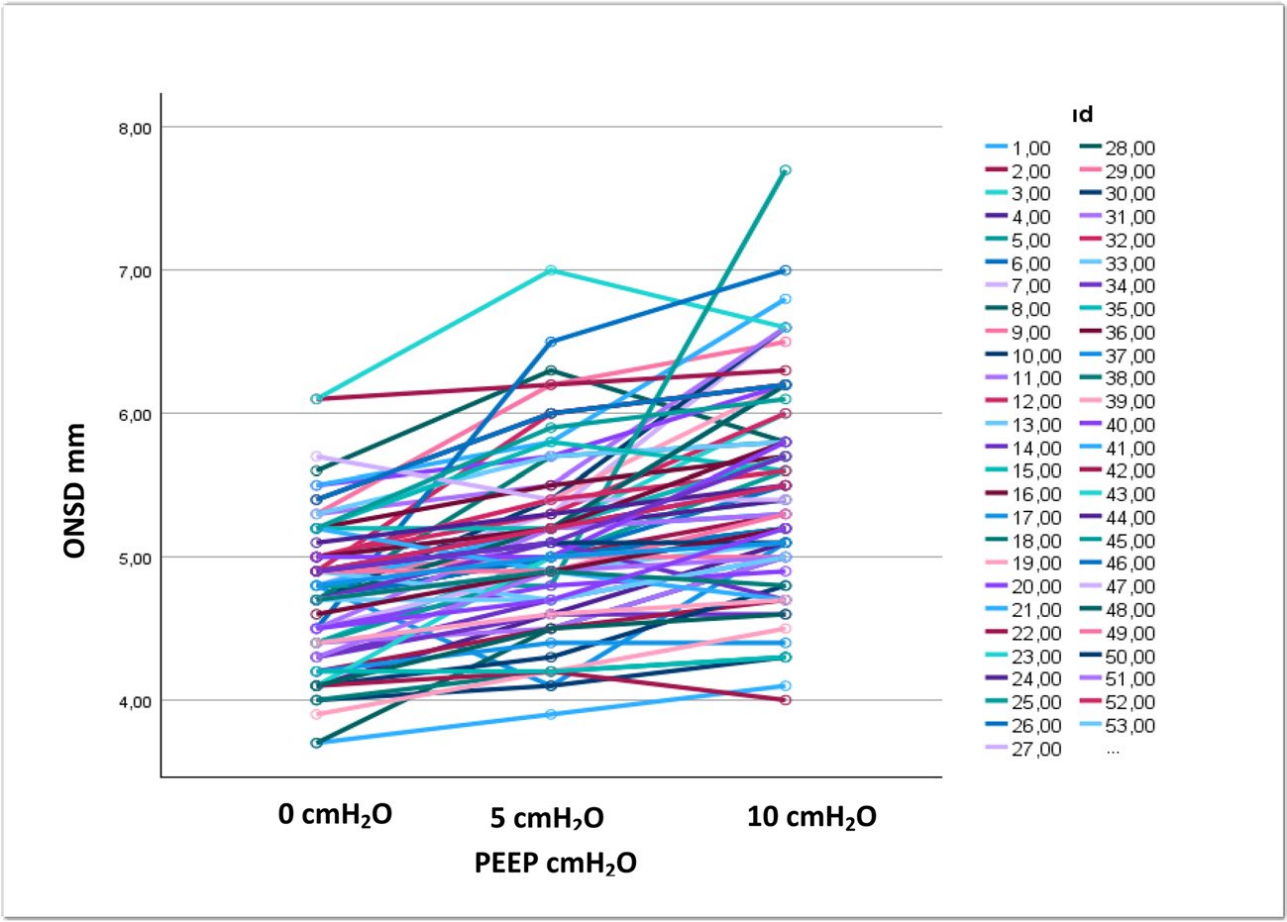
Distribution of ONSD in whole group of patients (*n* = 80)

**Fig. 4:**
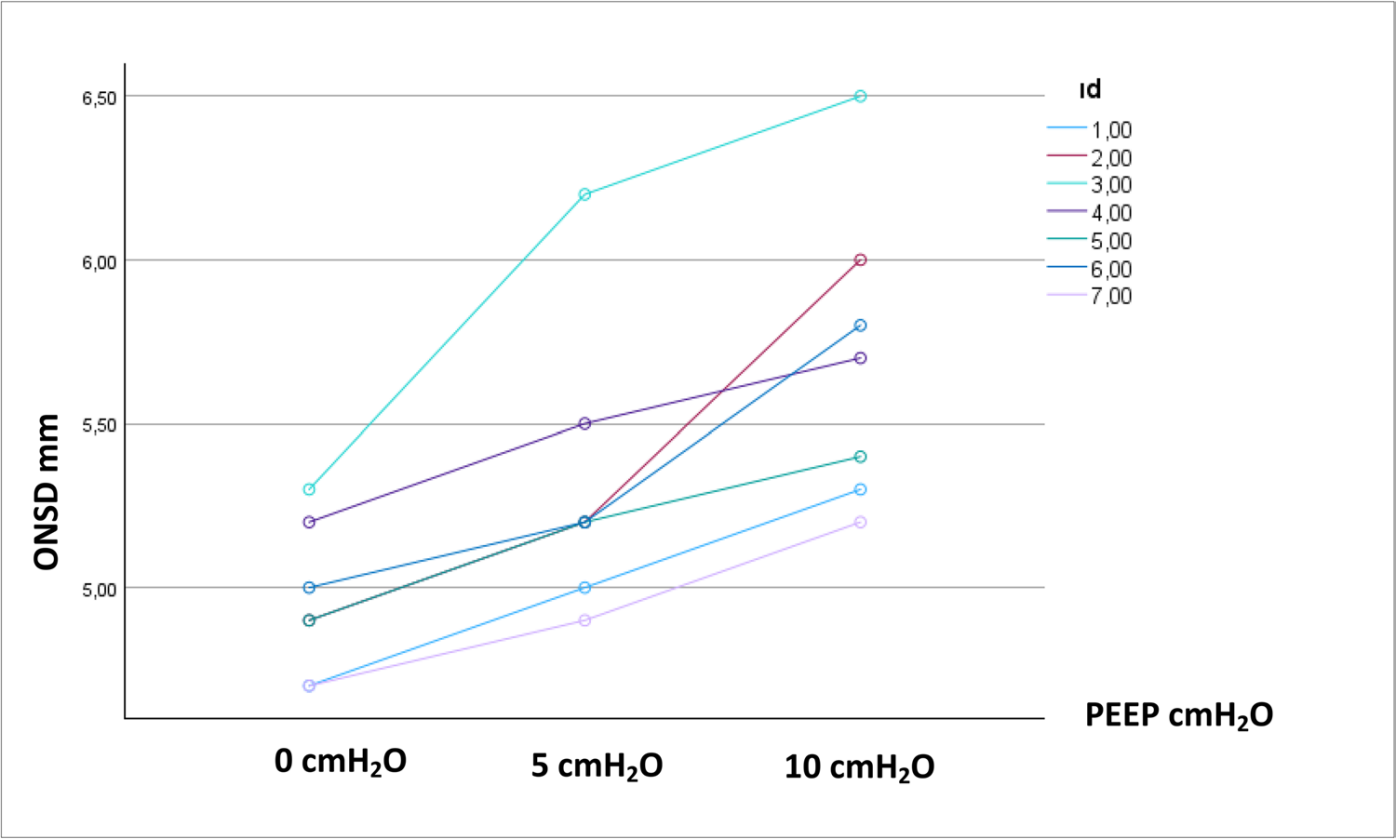
Distribution of ONSD in patients with intracranial midline shift (*n* = 7)

Distribution of ONSD in the whole group of patients and in patients with intracranial midline shift is shown in Figs. 3 and 4.

When we compared the effect of increasing PEEP value on ONSD in patients with different intracranial pathologies (supratentorial tumor, aneurysm, arteriovenous malformation, and epilepsy), no difference was found among the groups (*p* > 0.5, Table 5).

**Table 5:** Change of ONSD in patients with distinct type of intracranial pathology

	Supratentorial tumor *n* = 62		Aneurysm *n* = 8		Arteriovenous malformation *n* = 6		Epilepsy *n* = 4		*p
	Mean	Std. dev	Mean	Std. dev	Mean	Std. dev	Mean	Std. dev	
ONSD when PEEP 0 cmH_2_O (mm)	4.80 b	.48	4.80 b	.50	4.54 b	.56	4.65b	.33	.*508*
ONSD when PEEP 5 cmH_2_O (mm)	5.14 ab	.58	5.14 ab	.71	4.85 ab	.58	4.90 ab	.29	.*532*
ONSD when PEEP 10 cmH_2_O (mm)	5.49 a	.71	5.35 a	.73	5.29 a	.90	5.13a	.33	.*672*
**p	.***001***		.***001***		.***001***		.***018***		*İnteraksiyon: 0*.*768*

^*^Repeated measures of ANOVA with the post hoc Bonferroni correction test

The mean peak airway pressure was determined as 20.91 ± 3.07 cmH_2_O during 10 cmH_2_O PEEP application.

## Discussion

The mean ONSD values were determined as 4.77 mm, 5.1 mm, and 5.44 mm when 0, 5, and 10 cmH_2_O PEEP applied respectively in the present study. The statistically significant increases in ONSD due to increase in PEEP level were determined. As ONSD’s cutoff range of 5.6 to 6.3 mm is the most specific for diagnosis of elevated ICP in adults [2], the critical mean value was not exceeded during 10 cmH_2_O PEEP application in patients undergoing supratentorial craniotomy. However, application of 10 cmH_2_O PEEP in patients with a midline shift increased the mean ONSD value to 5.73 mm. This value is roughly 0.1 mm higher than the lower limit of the ONSD cutoff value.

The optic nerve sheath is in direct continuity with the dura mater and cerebrospinal fluid (CSF). Because optic nerve sheath is distensible, a rise in ICP is associated with an increase in the ONSD [12]. The correlation with the ultrasonography guided ONSD measurement and invasive intracranial pressure measurements has been shown [9]. The diagnostic accuracy of ONSD for the detection of increased ICP in patients with traumatic brain injury was reshown with very new meta-analysis [4, 16]. That is why ultrasonography guided ONSD measurement technique is a simple, noninvasive, and fast option to assess ICP [1].

Providing optimum ventilation without causing further rise in ICP is mandatory in patients with intracranial pathology. Although the most common reason for secondary brain injury following intracranial pathology is hypoxemia, dealing with hypoxemia would be difficult in clinical practice [5]. Administration of high fraction of oxygen can cause absorption atelectasis that leads to worsening hypoxemia. On the one hand, application of PEEP increases mean airway pressure, prevents alveolar collapse, and improves lung compliance and oxygenation; on the other hand, high levels of PEEP application is avoided in patients undergoing craniotomy to prevent rise in ICP. But that approach would increase the risk of secondary brain injury especially in patients with low lung compliance and hypoxemia.

Recent studies showed that PEEP application may not cause a rise in ICP [11, 14]. In neurosurgical intensive care patients requiring ICP monitoring and mechanical ventilation, PEEP at 5 cmH_2_O did not significantly alter intracranial pressure. The clinical relevance of the intracranial pressure increase at PEEP levels of 10 and 15 cmH_2_O was found questionable [11].

Muench et al. [14] assessed the effect of up to 20 cmH_2_O PEEP on ICP, brain tissue oxygen tension, regional cerebral blood flow, mean arterial pressure (MAP), and cardiac output in patients with severe subarachnoid hemorrhage. Stepwise elevation of PEEP resulted in a significant decrease of MAP and regional cerebral blood flow but does not impair intracranial pressure. PEEP application may indirectly affect cerebral perfusion via its negative effect on macrohemodynamic variables. Therefore, following severe subarachnoid hemorrhage, a PEEP-induced decrease of MAP should be prevented to maintain cerebral perfusion. We applied up to 10 cmH_2_O PEEP in our patient’s population and two patients presented hypotension that is why they were excluded from the study. We also determined a statistically significant increase in ONSD due to increase in PEEP level.

Preserving hemodynamic stability is very important to preserve cerebral hemodynamics. In Kwak et al. [10] trial, the effect of high positive end-expiratory pressure on cerebral oxygen saturation (rSO_2_) during laparoscopic cholecystectomy was assessed. That found that application of PEEP with 10 cmH_2_O during CO_2_ pneumoperitoneum could preserve the rSO_2_ value and hemodynamic stability in patients undergoing laparoscopic cholecystectomy under propofol anesthesia.

Carricato A. et al. [3] investigated 21 comatose patients with severe head injury or subarachnoid hemorrhage receiving ICP monitoring who required mechanical ventilation and PEEP. They compared the effect of PEEP up to 12 cmH_2_O in patients with low lung and normal lung compliance and found that in patients with low lung compliance, PEEP has no significant adverse effect on ICP and cerebral and systemic hemodynamics. Our study was performed in patients who were undergoing elective craniotomy and none of them had lung disease. We did not record the compliance but the mean peak airway pressure was determined as 20.91 ± 3.07 cmH_2_O during 10 cmH_2_O PEEP application. Although we did not observe low lung compliance in our study population, 10 cmH_2_O PEEP application did not exceed the cutoff ONSD values in patients undergoing supratentorial craniotomy. On the other hand, application of 10 cmH_2_O PEEP in patients with a midline shift increased the mean ONSD value to 5.73 mm in our study population. This value is roughly 0.1 mm higher than the lower limit of the ONSD cutoff value. That is why PEEP application up to 10 cmH_2_O should be performed carefully in patients with intracranial midline shift and it should be kept in mind that other factors may further increase ICP, such as hyperthermia, hypercapnia, or hypoxemia.

Khandelwal et al. [8] assessed the effect of up to 5 cmH_2_O PEEP in pediatric traumatic brain injury patients. They determined the mean ONSD values as 3.1 mm, 3.3 mm, and 3.5 mm when 0, 3, and 5 cmH_2_O PEEP applications respectively. In our study, the determined mean ONSD values were 4.77 mm, 5.1 mm, and 5.44 mm when 0, 5, and 10 cmH_2_O PEEP applications respectively. That difference would be reasonable when comparing the pediatric and adult population.

This study has some limitations. Although we stopped PEEP incensement when MAP < 55 mmHg, we did not record PEEP and MAP correlation; besides this, we did not monitor cardiac output. Besides this, the relatively low number of patients with midline shift is another limitation. Additionally, the standard deviations were quite high in patients without midline shift undergoing PEEP 5 and 10 cmH_2_O; a large proportion of individual patients exceeded the ONSD cutoff value both at PEEP values of 5 and 10 cmH_2_O. We believe that this does suggest some safety concerns for individual patients at these PEEP values.

## Conclusion

The statistically significant increases in ONSD due to increase in PEEP level were determined. As the cutoff value for elevated ICP assessed by ONSD is between 5.6 and 6.3 mm in adults, the critical mean value can be exceeded during 10 cmH_2_O PEEP application in patients with a midline shift. Therefore, PEEP application up to 10 cmH2O should be performed carefully, especially in patients with intracranial midline shift.

## Data Availability

We obtained written consent before data collections.
